# The Prophylactic Effect of Ivermectin Treatments on Nematode Infections of Mammals in a Faunistic Park (Northern Italy)

**DOI:** 10.3390/ani12091124

**Published:** 2022-04-27

**Authors:** Sergio A. Zanzani, Luca Villa, Alessia L. Gazzonis, Daniel Cartagena, Michele Mortarino, Eleonora Bonacina, Davide Guadagnini, Carolina Allievi, Maria Teresa Manfredi

**Affiliations:** 1 Department of Veterinary Medicine and Animal Sciences, Università Degli Studi di Milano, Via Dell’ Università 6, 26900 Lodi, Italy; luca.villa@unimi.it (L.V.); alessia.gazzonis@unimi.it (A.L.G.); michele.mortarino@unimi.it (M.M.); carolina.allievi@unimi.it (C.A.); mariateresa.manfredi@unimi.it (M.T.M.); 2 “Le Cornelle” Faunistic Park, Via Cornelle 16, 24030 Valbrembo, Italy; danielc94@hotmail.com (D.C.); bonacina@lecornelle.it (E.B.); oltolina@lecornelle.it (D.G.)

**Keywords:** parasitological monitoring, endoparasites, zoo mammals, nematode infections, ivermectin, FLOTAC

## Abstract

**Simple Summary:**

Nematode infections can pose a health risk to mammals housed in zoos and faunistic parks, where they live in environmental conditions far away from those of conspecifics in nature. To manage nematode infections, it is often necessary to adopt group prophylactic strategies by anthelmintic drugs. With the present study, it was possible to observe the effects of two prophylactic treatments with ivermectin adopted in a faunistic park in northern Italy and highlight the differences according to the different taxonomic groups of hosts and parasites.

**Abstract:**

Nematode infections of mammals can spread in zoos and faunistic parks and lead to disease in humans and animals. Group treatment strategies with anthelminthic drugs are common. Still, their effectiveness should be verified by sensitive and specific copromicroscopic analyses. This study assessed longitudinal parasitological monitoring, by FLOTAC^®^ dual technique, in mammals housed in an Italian faunistic park, in order to verify the effectiveness of the two adopted ivermectin prophylactic treatments. Twenty-one species of herbivorous mammals from ten families were treated twice per year with ivermectin in an in-feed formulation (medicated feed containing 1.7 g/ton ivermectin daily, for 30 days in March and November), while 13 species of carnivores and primates from five families were treated once a month with oral or subcutaneous administrations of ivermectin (200 μg/kg body weight (b.w.), from March to November). Fecal samples were collected in June–July and October 2019 (late spring–early summer and autumn sampling groups, respectively). All nematode infections, sustained by *Nematodirus* spp., *Capillaria* spp., *Trichuris* spp., *Parascaris* spp. and Strongylida, were detected in samples collected from herbivores, presenting prevalence rates of infection of 17.3% (9/52), 15.4% (8/52), 15.4% (8/52), 5.8% (3/52), and 3.8% (2/52), respectively. All carnivores and primates tested negative. The general linear mixed model showed that nematode eggs’ excretion in herbivores were influenced by sampling and sampling-host family interaction. Results showed that frequency and dose of prophylactic treatments in herbivores should be improved according to host and parasite taxonomic groups. The treatment adopted in carnivores and primates, together with hygienic management, was effective in nematode control.

## 1. Introduction

Mammals housed in zoos and faunistic parks deal with environmental and crowding conditions distinct from those of their wild conspecifics, supporting widespread nematode infections based on direct life cycles [1,2,3]. In these hosts, both imported species–specific parasites and nonspecific parasites occur [4,5,6]. Generally, nematode infections are asymptomatic in captive mammals; however, they can determine serious diseases or favor the onset of other pathologies [7,8,9,10,11]. Furthermore, a few nematode species that infect carnivores and primates can pose a risk to human health [12,13].

The control of parasitic nematodes in zoos and faunistic parks requires proper hygienic management, biosecurity plans, and group treatment strategies [14,15]. Among antiparasitic drugs, ivermectin, a macrocyclic lactone, is widely used in zoos and faunistic parks to control circulation of both nematodes and ectoparasites [16,17]. Its effectiveness has been verified in several species of mammals housed in zoos [18,19,20,21,22]. Despite its extensive use in such animals, few studies have monitored the effects of prophylactic treatments on the infection prevalence or egg fecal excretion by parasitic nematodes over time.

The present study aimed to evaluate the effects of two different strategies, based on prophylactic treatments with ivermectin, to control endoparasitic infection in two groups of zoo mammals. Therefore, a longitudinal parasitological monitoring method was planned in selected species of mammals housed in a faunistic park sited in northern Italy.

## 2. Materials and Methods

The study was carried out in a faunistic park sited in northern Italy (Latitude: 45°43′0.94″ N; Longitude: 9°35′50.16″ E) that housed 39 mammal species, in addition to 8 and 28 species of reptiles and birds, respectively. To control the circulation of endo- and ectoparasites, many mammals underwent prophylactic treatment with ivermectin according to their mammal groups [16]. Herbivores were treated twice/year (in March and November), daily for 15 days, with an in-feed ivermectin formulation (Ivomec^®^ Premix, Boehringer Ingelheim Animal Health Italia S.p.A, Milan, Italy). The medicated feed, administered ad libitum and containing 1.7 q/ton of Ivomec^®^ Premix (~10 g/ton ivermectin [19]), was produced for the faunistic park by a commercial feed mill (Agricola Italiana Alimentare S.p.A, Quinto di Valpantena, Italy). Carnivores and primates were treated once a month, from March to November, with oral or subcutaneous administrations of ivermectin (200 μg/kg b.w.; Ivomec^®^, Boehringer Ingelheim Animal Health Italia S.p.A, Milano, Italy), depending on animal behavior/compliance and operators’ safety. Thisparasitological study included 21 species of herbivores and 13 species of carnivores and primates. A comparison was made between these two prophylactic treatments because they involved most of the terrestrial mammals present in the faunistic park. Other species received targeted treatments for specific needs [23]. Individual or pooled fecal samples from these hosts were collected according to their housing (individual or in group); for grouped animals, single fecal masses were collected in plastic bags and pools were formed afterwards. Sampling was carried out in the morning with the assistance of animal keepers; cages, boxes, and enclosures where animals spent the night were cleaned the evening before sampling to ensure collection of fresh fecal samples. For both host groups, two samplings were performed from the second half of June through the first half of July 2019 (late spring–early summer sampling group) and in October 2019 (autumn sampling group). Overall, 52 (46 pooled samples and 6 individual samples) and 32 (28 pooled samples and 4 individual samples) fecal samples were collected from 153 herbivorous mammals and 28 carnivores and primates, respectively (Table 1 and Table 2). Animals that contributed to producing a certain pooled fecal sample were the same in both samplings.

Carnivore and primate fecal samples were all negatives in both samplings (Table 2).

Fecal samples were refrigerated and quantitative copromicroscopic exams were performed within 48 h by FLOTAC^®^ dual technique with an analytic sensitivity of two eggs/larvae/oocysts per gram (EPG/LPG/OPG) of feces [24,25,26]. To obtain pooled samples from grouped animals, the same amount of feces (possibly at least 5 g, depending on species size) was used from each fecal mass of the group, and pooled feces were homogenized according to the FLOTAC^®^ user manual. The flotation solutions FS2 (NaCl; s.g. = 1200) and FS7 (ZnSO_4_; s.g. = 1350)—useful for the detection of nematode, cestode and trematode eggs, nematode larvae and coccidian oocysts—were employed to process all the collected samples.

The percentage of samples testing positive for nematode eggs was calculated and then compared in herbivores treated twice/year and in carnivores and primates treated monthly by chi-square test. To identify any association between nematode eggs’ excretion and selected variables, logarithmically transformed nematode EPG values (Log(EPG+1)) of each sample were introduced as the dependent variable in a generalized linear mixed model (GLMM) with repeated measures. Host family, time of sampling (late spring–early summer vs. autumn), and their interaction were introduced as independent categorical variables in the model. The identity of each fecal sample was included as a random intercept effect. The final model was determined by backward elimination of nonsignificant variables (*p* ≥ 0.05) and best corrected Akaike information criteria (AIC). Statistical analyses were implemented by SPSS 20.0 (IBM, Chicago, IL, USA).

## 3. Results

Out of 84 fecal samples, 23 (27.4%, 95% Confidence Interval (CI): 18.2–38.2) were positive for at least one parasite taxon. Nematode larvae and cestode and trematode eggs were not found in any samples. The percent positivity was higher in autumn sampling (33.3%, 95% CI: 19.6–49.6; 14/42) than the late spring–early summer sampling (21.4%, 95% CI: 10.3–36.8; 9/42). In herbivores, the percentage of positivity was 40.4% (95% CI: 27–54.9; 21/52; Table 1).

This differences in the percentage of infections were highly significant when compared by chi-square test (Pearson’s chi-square = 17.231; *p*-value = 0.00003). In herbivores, the following taxa were identified in fecal samples: *Nematodirus* spp. (17.3%, 95% CI: 8.2–30.3; 9/52), *Capillaria* spp. (15.4%, 95% CI: 6.8–28.1; 8/52), *Trichuris* spp. (15.4%, 95% CI: 6.8–28.1; 8/52), *Parascaris* spp. (5.8%, 95% CI: 1.2–16; 3/52), and Strongylida (3.8%, 95% CI: 0.5–13.2; 2/52). EPG values in positive samples ranged from two to 578. *Eimeria* spp. oocysts were also detected (13.5%, 95% CI: 5.6–25.8; 7/52) (Table 1).

Since carnivores and primates all tested negative, GLMM was only implemented for copromicroscopic data from herbivores. In the final model, time of sampling and interaction time of sampling × host family were significant predictors of the logarithmically-transformed nematode EPG (Table 3).

**Figure 1:**
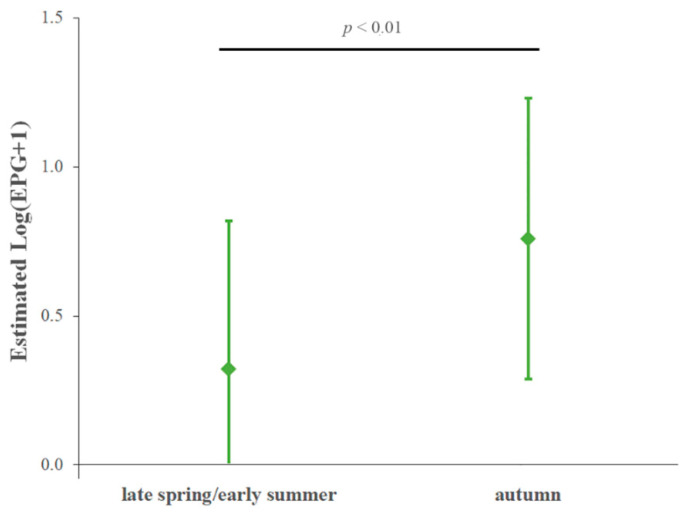
Estimated nematode Log(EPG+1), by time of sampling, in herbivores housed in the studied faunistic park obtained by a generalized linear mixed model. Vertical bars: 95% confidence intervals; horizontal black bar: pairwise comparison between nematode eggs excretion in the two sampling points.

Values of logarithmically-transformed nematode EPG estimated by the model were significantly higher in the autumn sampling than the late spring–early summer sampling (*p*-value < 0.01) (Figure 1).

**Figure 2:**
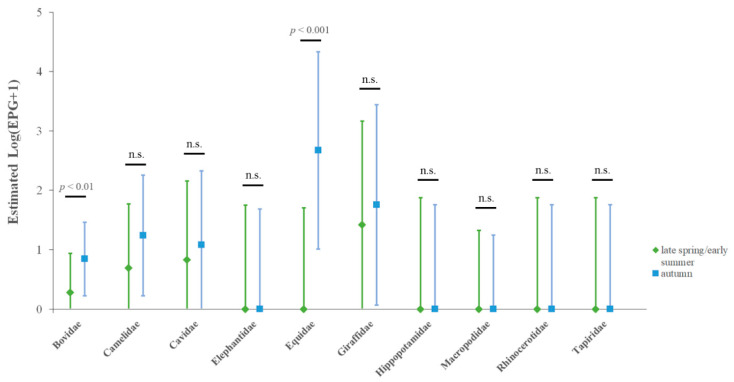
Estimated nematode Log(EPG+1) by host family and time of sampling in herbivores housed in the studied faunistic park, obtained by a generalized linear mixed model. Vertical bars: 95% confidence intervals; horizontal black bar: significant pairwise comparison between nematode eggs excretion in the two samplings by family; n.s.: not significant.

Estimated nematode Log(EPG+1) of the two sampling points also differed by host family; pairwise comparisons showed significant differences between the two sampling points in two out of 10 herbivores families (Figure 2).

Particularly, in Bovidae family, estimated Log(EPG+1) were 0.28 and 0.84 in late spring–early summer and autumn samplings, respectively (*p* < 0.01); in Equidae family, estimated Log(EPG+1) were 0 and 2.67 in late spring–early summer and autumn samplings, respectively (*p* < 0.001).

## 4. Discussion

In the studied faunistic park, 181 mammals belonging to 15 different families received an anthelmintic prophylactic treatment with ivermectin. Nematode infections were detected only in herbivores that received a twice/year prophylactic treatment with an in-feed ivermectin formulation, while the carnivores and primates that received a monthly prophylactic treatment from March to November were negative in both samplings. Moreover, the implemented GLMM showed that the overall nematode eggs’ excretion increased in the autumn sampling.

In herbivores, Strongylida eggs were identified only in two samples, and the egg excretion detected was very low. The circulation of these parasites seemed to be lower when compared to other Italian and European studies [1,2,27,28,29]. However, they were similar to what was observed by Pérez Cordon et al. [30] in a Spanish zoological garden, where Strongylida were not found; management and prophylactic treatments administered in the studied faunistic park seemed to be effective at controlling the circulation of gastrointestinal strongyles.

As regards the other detected nematode taxa (*Nematodirus* spp., *Capillaria* spp., *Trichuris* spp., *Parascaris* spp.), all of them showed higher percentages of infection and EPG values than Strongylida. The reasons why these parasites circulated more than Strongylida could be different. First, environmental resistance to parasites’ free-living stages in paddocks with scarce grass cover must be considered. It could be hypothesized that, during the 30 days of prophylactic treatment with the in-feed ivermectin formulation, free-living stages of Strongylida did not find environmental conditions sufficient to survive and reinfect hosts, even if the prophylactic treatment was interrupted [31]. On the other hand, eggs of *Nematodirus* spp., *Capillaria* spp., *Trichuris* spp., and *Parascaris* spp., all having higher environmental resistance, probably persisted longer in the soil. To control the circulation of these parasite genera with resistant eggs, a more frequent administration or the use of anthelmintic formulations less prone to underdosing (e.g., oral or pour-on solutions for individual treatment) could be needed, together with suitable management strategies aiming to reduce soil contamination.

Furthermore, data obtained in this study suggested that the anthelmintic efficacy of the prophylactic treatment should be specifically investigated for some nematode taxa in certain host species by fecal egg count reduction test (FECRT), following the World Association for the Advancement of Veterinary Parasitology guidelines [32,33]. Considering *Parascaris* spp. infections in Equidae, we postulated that the March ivermectin prophylaxis was effective without performing an FECRT. In fact, despite the presence of the parasite in the faunistic park, the three late spring–early summer fecal samples collected from *Equus quagga* tested negative (0 EPG) for nematodes within the 3-month prepatent period [34]. After this period, reinfections by embryonated eggs in soil determined the results of the autumn sampling; all samples tested positive for *Parascaris* spp., presenting an average value of 587 EPG. On the contrary, the late spring–early summer sampling fell outside (or borderline to) the prepatent period of *Nematodirus* spp., *Capillaria* spp. and *Trichuris* spp. Thus, without performing a rigorous FECRT, it was impossible to say whether samples tested positive for reinfection or it was a lack of efficacy of the treatment. It should also be considered that certain genera or species of parasites can represent the limiting taxonomic group for the dosage of an anthelmintic active ingredient [35]; therefore, the effectiveness of this prophylaxis should be tested on each one. However, the results of GLMM suggested that there was at least a partial efficacy of the treatment against these parasites. In fact, starting from a lower EPG level of the late spring–early summer sampling, closer to the March prophylactic treatment, the nematode EPG increased for reinfections in the autumn sampling.

It is probable that the effects of prophylactic treatments against nematode circulation in the herbivores housed in the faunistic park could depend, not only on parasites’ life cycle or their susceptibility to drugs, but also on the host species. Indeed, the implemented GLMM showed that seasonal increases in nematode eggs excretion differed by host family. This result could have several explanations. First of all, different hosts may have different susceptibility to parasites able to circulate within the studied faunistic park. Indeed, Elephantidae, Hippopotamidae, Macropodidae, Rhinocerotidae, and Tapiridae were not suitable hosts for the *Nematodirus* spp., *Capillaria* spp., and *Trichuris* spp. that infected other animals housed in the park [36,37,38,39].

A few differences could also be attributed to physiology and metabolism between infected hosts. In domestic ruminants, it is well known that the detoxifying capacities toward xenobiotics are more significant in goats than in sheep, as a consequence of their feeding behavior [40]. The same could apply to herbivores housed in faunistic parks. In nature, Giraffidae are considered general browsers [41] and are probably more exposed to plant toxins. For these animals, the absence of a significant difference in nematode EPG between the late spring–early summer and autumn samplings could be due to the rapid detoxification of the administered ivermectin. Therefore, administration of specific dosages of anthelmintics should be further evaluated in different taxonomic groups of herbivores housed in faunistic parks, as required for goats when compared with sheep [42]. Possible underdosing due to both parasite (limiting taxonomic group for the dosage of an active anthelmintic ingredient) and host features could also determine the development of anthelmintic resistance, mainly when only one anthelminthic family is repeatedly used for treatments. The alternated or combined use of other anthelmintics (i.e., fenbendazole), belonging to different families, could be useful to slow down resistance development [43].

Nematode infections were not detected in any samples collected from carnivores and primates treated monthly with oral or subcutaneous ivermectin. Thus, this prophylactic treatment seemed to be particularly effective at controlling nematode circulation in the studied faunistic park. In other European studies, carnivores and primates of faunistic parks were infected by several nematode taxa (i.e., *Toxocara* spp., *Toxascaris* spp., *Ancylostoma* spp., *Uncinaria* spp., *Strongyloides* spp., *Ascaris* spp., *Enterobius* spp., *Trichuris* spp., Strongylida), often of zoonotic concern [1,2,3,24,26]. Proper management and prophylactic treatments are highly recommended to avoid circulation of those nematodes that pose a risk to animal and human health.

The parasitological monitoring carried out in the present study was not without limits. We were unable to determine with certainty the efficacy of the prophylactic treatments against all the parasitological taxa detected. In the future, it would be advisable to verify their effectiveness by FECRT. Furthermore, the parasitological negativity observed in carnivores and primates should be confirmed in the winter period, during which they do not receive the prophylactic treatment.

## 5. Conclusions

Circulation of nematodes in zoos and faunistic park poses a risk for the health of humans and animals. Results obtained in the present study showed that parasitological monitoring of animals housed in faunistic parks could provide both information on the efficacy of prophylactic treatments adopted and indications to limit or avoid parasite circulation. Considering the low EPG/OPG excretion detected in several samples in the present survey, parasitological monitoring should be conducted with sensitive and specific techniques, to collect the most detailed information possible. Efficacy of the adopted hygiene management and prophylactic treatments should be verified, both to further reduce the risk of nematode infection and to calibrate anthelmintic drug administration. Unsatisfactory protocols for frequency and dosage should be improved, and the use of more than one pharmacological family could be considered. Effective control of zoonotic nematodes is important; thus, methods to increase the effectiveness of available treatments should be considered.

## Figures and Tables

**Table 1:** Endoparasites detected by quali/quantitative copromicroscopic analyses (FLOTAC^®^ dual technique) in herbivores from a faunistic park in northern Italy. When more than one sample for species is tested, EPG/OPG is the mean value.

Family	*Species*	N. of Animals	N. of Fecal Samples	1st Sampling (Late Spring/Early Summer)		2nd Sampling (Autumn)	
				N. of Positives/Sampled	Detected Parasites (EPG/OPG)	N. of Positives/Sampled	Detected Parasites (EPG/OPG)
Bovidae							
*Antilope cervicapra*		10	1	1/1	*Nematodirus* spp. (20)	1/1	*Nematodirus* spp. (26)
*Kobus leche*		10	1	0/1	--(0)	0/1	--(0)
*Kobus megaceros*		11	1	0/1	--(0)	0/1	--(0)
*Oryx dammah*		4	1	1/1	*Eimeria* spp. (126)	1/1	Strongylida (2) *Capillaria* spp. (32) *Eimeria* spp. (244)
*Ovis aries*		20	1	1/1	*Nematodirus* spp. (4) *Eimeria* spp. (46)	1/1	*Trichuris* spp. (8) *Eimeria* spp. (134)
*Taurotragus oryx*		2	1	0/1	--(0)	0/1	--(0)
*Tragelaphus eurycerus*		3	2	1/2	*Capillaria* spp. (5)	2/2	*Capillaria* spp. (46)
*Tragelaphus spekii*		7	1	0/1	--(0)	1/1	*Capillaria* spp. (8)
Camelidae							
*Camelus bactrianus*		3	1	1/1	Strongylida (2) *Nematodirus* spp. (4) *Trichuris* spp. (110) *Eimeria* spp. (14)	1/1	*Trichuris* spp. (578) *Eimeria* spp. (112)
*Lama glama*		3	1	0/1	--(0)	1/1	*Nematodirus* spp. (8)
*Vicugna pacos*		3	1	0/1	--(0)	0/1	--(0)
Cavidae							
*Cavia porcellus*		40	1	1/1	*Eimeria* spp. (40)	0/1	--(0)
*Dolichotis patagonum*		5	1	1/1	*Capillaria* spp. (44)	1/1	*Capillaria* spp. (142) *Trichuris* spp. (2)
Elephantidae							
*Elaphas maximus*		2	2	0/1	--(0)	0/1	--(0)
Equidae							
*Equus quagga*		5	3	0/3	--(0)	3/3	*Parascaris* spp. (587)
Giraffidae							
*Giraffa camelopardalis*		7	2	2/2	*Nematodirus* spp. (64) *Trichuris* spp. (6)	2/2	*Nematodirus* spp. (5) *Capillaria* spp. (1) *Trichuris* spp. (50)
Hippopotamidae							
*Hippopotamus amphibius*		3	1	0/1	--(0)	0/1	--(0)
Macropodidae							
*Macropus rufogriseus*		6	1	0/1	--(0)	0/1	--(0)
*Macropus rufus*		5	1	0/1	--(0)	0/1	--(0)
Rhinocerotidae							
*Diceros bicornis*		3	1	0/1	--(0)	0/1	--(0)
Tapiridae							
*Tapirus terrestris*		1	1	0/1	--(0)	0/1	--(0)
TOTAL		153	26	9/26	--	14/26	--

N. = number.

**Table 2:** Endoparasites detected by quali/quantitative copromicroscopic analyses (FLOTAC^®^ dual technique) in carnivores and primates from a faunistic park in northern Italy.

Family	*Species*	N. of Animals	N. of Fecal Samples	1st Sampling (Late Spring/Early Summer)		2nd Sampling (Autumn)	
				N. of Positives/Sampled	Detected Parasites (EPG/OPG)	N. of Positives/Sampled	Detected Parasites (EPG/OPG)
Cebidae							
*Saguinus oedipus*		4	1	0/1	--(0)	0/1	--(0)
*Saimiri sciureus*		4	1	0/1	--(0)	0/1	--(0)
Felidae							
*Neofelis nebulosa*		2	1	0/1	--(0)	0/1	--(0)
*Panthera leo*		2	1	0/1	--(0)	0/1	--(0)
*Panthera pardus*		2	1	0/3	--(0)	0/3	--(0)
*Panthera tigris*		3	3	0/3	--(0)	0/3	--(0)
*Panthera uncia*		2	1	0/1	--(0)	0/	--(0)
*Puma concolor*		1	1	0/1	--(0)	0/1	--(0)
Hyaenidae							
*Hyaena hyaena*		1	1	0/1	--(0)	0/1	--(0)
Hylobatidae							
*Hylobates lar*		6	1	0/1	--(0)	0/1	--(0)
*Symphalangus syndactylus*		5	2	0/2	--(0)	0/2	--(0)
Lemuridae							
*Lemur catta*		6	1	0/1	--(0)	0/1	--(0)
*Varecia variegata*		3	1	0/1	--(0)	0/1	--(0)
TOTAL		28	16	0/16	--	0/16	--

N. = number.

**Table 3:** Effect of selected risk factors on nematode fecal egg count (logarithmically transformed) in herbivores housed in the studied faunistic park, obtained by a generalized linear mixed model. In bold: significant predictors of Log(EPG+1).

Independent Variables	F	Degrees of Freedom	*p*-Value
Time of sampling	9.566	1	**0.004**
Host family	0.698	9	0.706
Time of sampling × host family	5.068	9	**<0.0005**

## Data Availability

The datasets used and analyzed during the current study are available from the corresponding author on reasonable request.
